# The Toxicity Assessment of Iron Oxide (Fe_3_O_4_) Nanoparticles on Physical and Biochemical Quality of Rainbow Trout Spermatozoon

**DOI:** 10.3390/toxics6040062

**Published:** 2018-10-18

**Authors:** Mustafa Erkan Özgür, Ahmet Ulu, Sevgi Balcıoğlu, İmren Özcan, Süleyman Köytepe, Burhan Ateş

**Affiliations:** 1 Department of Aquaculture, Faculty of Fishery, Malatya Turgut Özal University, Malatya 44280, Turkey; 2 Department of Chemistry, Science Faculty, İnönü University, Malatya 44280, Turkey; ahmet.ulu@inonu.edu.tr (A.U.); sevgi.balcioglu@gmail.com (S.B.); imrenozcan@gmail.com (İ.Ö.); suleyman.koytepe@inonu.edu.tr (S.K.); burhan.ates@inonu.edu.tr (B.A.)

**Keywords:** Fe_3_O_4_ nanoparticles, *Oncorhynchus mykiss*, spermatozoon kinematics, oxidative stress biomarkers

## Abstract

The aim of this study was to evaluate the in vitro effect of different doses (50, 100, 200, 400, and 800 mg/L) of Fe_3_O_4_ nanoparticles (NPs) at 4 °C for 24 h on the kinematics of rainbow trout (*Oncorhynchus mykiss*, Walbaum, 1792) spermatozoon. Firstly, Fe_3_O_4_ NPs were prepared at about 30 nm from Iron (III) chloride, Iron (II) chloride, and NH_3_ via a co-precipitation synthesis technique. Then, the prepared Fe_3_O_4_ NPs were characterized by different instrumental techniques for their chemical structure, purity, morphology, surface properties, and thermal behavior. The size, microstructure, and morphology of the prepared Fe_3_O_4_ NPs were studied by Fourier transform infrared spectroscopy (FTIR), X-ray diffraction (XRD) spectroscopy, and scanning electron microscopy (SEM) equipped with an energy-dispersive X-ray spectrometer (EDS). The thermal properties of the Fe_3_O_4_ NPs were determined with thermogravimetric analysis (TGA), differential thermal analysis (DTA), and differential scanning calorimeter (DSC) analysis techniques. According to our results, there were statistically significant (*p* < 0.05) decreases in the velocities of spermatozoon after treatment with 400 mg/L Fe_3_O_4_ NPs. The superoxide dismutase (SOD) and catalase (CAT) activities were significant (*p* < 0.05) decrease after 100 mg/L in after exposure to Fe_3_O_4_ NPs in 24 h. As the doses of Fe_3_O_4_ NPs increases, the level of malondialdehyde (MDA) and total glutathione (tGSH) significantly (*p* < 0.05) increased at doses of 400 and 800 mg/L.

## 1. Introduction

In today’s technology, magnetic nanoparticles have an increasing importance [1,2,3]. In particular, Fe_3_O_4_ is widely used in advanced technological applications such as magnetic imaging, drug release systems, enzyme immobilization matrices, catalyst support materials, cell separating molecules, hyperthermia, and reinforcement for some composites. Recently, many papers have reported on the implications and applications of Fe_3_O_4_ nanoparticles (NPs), which are termed magnetite. Magnetite exhibits outstanding physicochemical properties due to the presence of both Fe(II) and Fe(III) in its structure. In particular, it behaves as a super paramagnetic when the particle size is reduced to a few nanometers [4]. Iron-based NPs have been used in many applications such as the treatment of chlorinated solvents and metals, the prevalent application for soil and groundwater remediation, biomedical applications (magnetic resonance imaging, drug delivery, and cell labeling), the treatment of water adsorption capacity, to improve surface modification, and in protective shells, solid supports, and the doping of a second metal [5]. For instance, Naha et al. showed that dextran-coated bismuth-Fe_3_O_4_ NPs could be utilized as contrast agents for computed tomography and magnetic resonance imaging [6]. Similarly, several groups synthesized Fe_3_O_4_ NPs-loaded polymeric microbubbles to create a bimodality platform; they also investigated the use of these particles as multimodal contrast agents [7,8]. In addition, it has been reported that Fe_3_O_4_ NPs can be used to both control plaque-biofilms and prevent dental caries since they have an intrinsic enzyme mimetic activity similar to natural peroxidases [9,10]. As a result of these wide applications, Fe_3_O_4_ NPs are also increasingly released in the environment. Furthermore, the potential ecotoxicological impacts in aquatic environments have aroused increasing attention. For example, over the last five years, there have been some studies about the ecotoxicology of iron-based NPs in aquatic animals such as an Indian major carp fish (*Labeo rohita*) [11], tilapia (*Oreochromis niloticus*) [12], Chinook salmon (*Oncorhynchus tshawytscha*) [13], zebrafish (*Danio rerio*) [14,15,16], *Mytilus galloprovincialis* [17], *Daphnia magna* [18], *Artemia salina* [19,20], and rotifer (*Brachionus rotundiformis*) [21].

In vitro methods allow the determination of the mechanisms and areas of action of pollutants by applying a wide range of exposure times and concentrations to compare the toxicity levels of environmental pollutants [22]. However, there are not many studies on in vitro toxicity of Fe_3_O_4_ NPs in fish sperm. Therefore, in this study we aimed to determine the in vitro toxicity of Fe_3_O_4_ NPs and its effects on the kinematics and oxidative stress markers of spermatozoon in rainbow trout (*Oncorhynchus mykiss,* Walbaum 1792). Thus, we aimed to test and understand the effects of this toxicity on the reproductive system of fish in aquatic ecology.

## 2. Materials and Methods

### 2.1. Instrumentation and Reagents

The chemicals used for nanoparticle synthesis and toxicological studies were all of high purity. FeCl_3_·4H_2_O, FeCl_2_·4H_2_O, and NH_3_ were obtained from a distributor of Merck Co. in Turkey. All chemicals used for toxicological studies were obtained from Sigma-Aldrich Co. (Saint Louis, MO, USA). All other chemicals were of the highest purity and commercially available and all solutions were prepared with distilled water.

Fourier transform infrared spectroscopy (FTIR) (Mattson 1000) was used to characterize the chemical structure of prepared Fe_3_O_4_ NPs. The thermal properties of the prepared Fe_3_O_4_ NPs were determined using TGA-50 (Shimadzu, Kyoto, Japan) and DTA-50 (Shimadzu, Kyoto, Japan) under a static air atmosphere and at a heating rate of 10 °C min^−1^ in the temperature range from 30 to 1000 °C. The DSC measurements of the Fe_3_O_4_ NPs were performed on a DSC-60 (Shimadzu, Kyoto, Japan). All samples (about 5 mg) were placed in sealed aluminum pans before heating under nitrogen flow (20 mL/min) at a scanning rate of 10 °C/min. An Al_2_O_3_ (5 mg)-filled aluminum crucible was used as a reference. The surface structure and morphological properties of the obtained Fe_3_O_4_ NPs were investigated with SEM-EDX (LEO Evo-40 VPX). The Fe_3_O_4_ NPs were characterized by XRD for the crystal structure and impurity. A Rigaku Rad B-Dmax II powder X-ray diffractometer was used for the XRD patterns of these samples. The 2*θ* values were taken from 2° to 85° with a step size of 0.04° using Cu Kα radiation (*λ* value of 2.2897 Å).

### 2.2. Synthesis of Fe_3_O_4_ NPs

In the study, FeCl_2_·4H_2_O (2 g) was added to 50 mL of distilled water and mixed for 1 h. Then, FeCl_3_·4H_2_O (5.45 g) was added to 50 mL of water in another flask, and stirred for 1 h. At the end of the 1-h mixing time, 50 mL of oleic acid was added to each of the solutions in each portion and mixed for 30 min. After that, the first and second portions of the solutions were taken into a 250 mL balloon and mixing was continued for 30 min. Then, 20 mL of 1.5 M NH_3_ solution was added dropwise. The mixture was stirred at room temperature for 1 day with a mechanical stirrer and a light brownish material was obtained at the end of the reaction. The product was centrifuged and washed with ethanol four times.

### 2.3. Collection and Exposure of Sperm Samples

The rainbow trout males (1850 ± 110 g) were maintained in the hatchery station at a commercial fish farm, Malatya, Turkey. Sperm samples were obtained in January 2018. Stripping was performed by massage from the front to the back of the fish abdomen without anesthesia. Fresh sperm samples were diluted with immotile solution (IMS) and activated by motile solution (MS). Immotil solution (IMS) was prepared by NaCl, 103 mmol/L; KCl, 40 mmol/L; CaCl_2_, 1 mmol/L; MgSO_4_, 0.8 mmol/L; Hepes, 20 mmol/L; 1000 mL of distilled water; pH 7.8 as a stock solution [23]. Motil solution (MS) was prepared by CaCl_2_ 1 mM; Tris 20 mM, Glycine 30 mM, NaCl 125 mM; 1000 mL of distilled water; pH 9 [24,25].

The pooled sperm samples, taken from six individual fish, were exposed to Fe_3_O_4_ NPs. The pooled sample was diluted with IMS to obtain a spermatozoon density of about 13 × 10^8^ cells/mL. The exposure was conducted with nominal concentrations such as 50, 100, 200, 400, and 800 mg/L of Fe_3_O_4_ NPs at 4 °C for 24 h in Eppendorf tubes. Sperm samples were first diluted at the ratio of 1:100 with IMS solution. The sperm samples were activated with motile solution (MS) at the ratio of 1:20 under the microscope. Final dilution rate was 2000 times.

### 2.4. Determination of Biochemical Oxidative Markers

For sperm samples preparation for a biochemical assay, each sample was sonified with an ultrasonifier (Bandel in Sonopuls HD 2070) in phosphate buffer solution (PBS) following a previously published protocol [26]. Afterward, the homogenates were centrifuged at 10,000 rpm for 10 min at 4 °C and the supernatants were separated for further analysis. For the measurement of antioxidant enzymes activity, the catalase (CAT) activity was measured by following the reduction of hydrogen peroxide (H_2_O_2_) at 240 nm at room temperature [27]. The CAT activity was then calculated according to the rate of the change in absorbance and expressed in U/mg protein. One unit of CAT represents the amount of enzyme that decomposes 1 μmol of H_2_O_2_ per minute.

Superoxide dismutase (SOD) activity was determined using the xanthine oxidase/cytochrome C method [28]. One unit of SOD activity is the amount of enzyme required to cause a half-maximal inhibition of cytochrome C reduction. Results were expressed in U/mg protein.

The total glutathione (tGSH) was determined spectrophotometrically [29] using 5,5′-Dithiobis (2-nitrobenzoic acid) (DTNB) at 412 nm. This colorimetric assay is based on the reaction between glutathione (GSH) and DTNB where TNB (5-thio-2-nitrobenzoic acid) is formed. A standard curve was prepared with known amounts of GSH. The values were expressed in nmol/mg protein.

Lipid peroxidation was measured by using the thiobarbituric acid (TBA) solution for malondialdehyde (MDA). The solution was added to the semen samples and the reaction mixture was incubated at 100 °C for 30 min. The samples were cooled and centrifuged at 14,000 rpm for 10 min. Then the resulting supernatant was separated and the absorbance of the supernatant was taken at 440 nm at room temperature using an ELISA microplate reader (Biotek, Winooski, VT, USA). The level of MDA was expressed as nmol MDA/mg protein. 

Protein was estimated according to the method reported by Bradford using bovine serum albumin (BSA) as a standard [30].

### 2.5. Determination of Spermatozoon Kinematics

After 24 h, the samples were examined under an Olympus CX31 microscope with a 200× magnification lens and a Sony CCD camera with 30 fbs. Spermatozoon velocity parameters such as VSL: straight line velocity (μm/s), VCL: curvilinear velocity (μm/s), and VAP: angular path velocity (μm/s), as well as movement style parameters such as LIN: linearity (%), the ratio of net distance moved to total path distance, BCF: beat cross frequency turning points of the spermatozoon head (Hz) and ALH: amplitude of lateral displacement of the spermatozoon head (μm) [31] were carried out by the computer-assisted sperm analysis systems, BASA-Sperm Aqua, produced by Merk Biotechnology Ltd. Co. in Turkey.

### 2.6. Statistics

Descriptive analysis (Means ± SE, *p* < 0.05) in Univariate Variance (two way-ANOVA) and Multiple Variance Analysis (MANOVA) with the Duncan test were used between groups after the homogeneity of each group was tested through the Test of Homogeneity of Variance in the SPSS 17 program. The graphics were created by Graph Pad Prism 5.

## 3. Results

### 3.1. Characterization of Fe_3_O_4_ NPs

**Figure 1:**
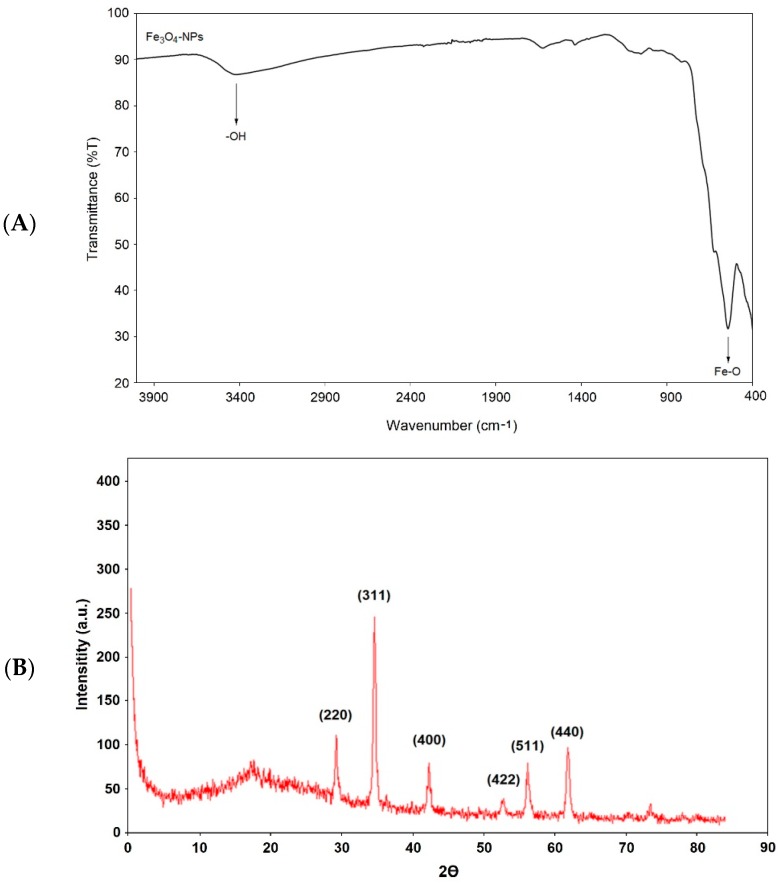
FTIR spectrum (**A**), X-ray spectrum (**B**) of Fe_3_O_4_ NPs structures.

**Figure 2:**
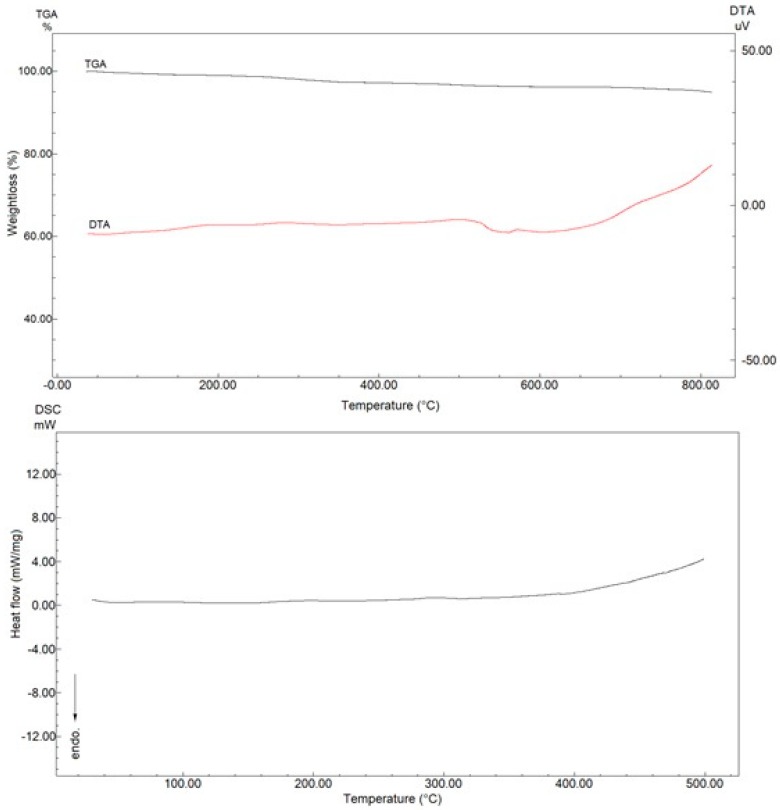
TGA, DTA and DSC thermograms of Fe_3_O_4_ NPs structures.

The FTIR spectrum for Fe_3_O_4_ NPs structures is given in Figure 1A. In this figure, the hydrogen bond tension of the -OH groups on the surface of the nanoparticles is seen between 3300 and 3600 cm^−1^. Moreover, the peak at 600 cm^−1^ is due to the Fe-O stretching vibration. All these peaks demonstrate the desired Fe_3_O_4_ NPs chemical structure [32]. The X-ray spectrum of the synthesized Fe_3_O_4_ NPs was also monitored, and is shown in Figure 1B. In this spectrum, 220 peaks at 29.3°, 311 peaks at 35.1°, and 400 peaks at 43.4° can be seen [33]. In addition, 422 peaks at 54.3°, 511 peaks at 56.7°, and 440 peaks at 62.7° confirm the Fe_3_O_4_ NPs structure [34]. The obtained Fe_3_O_4_ structures were obtained as pure and clean, as seen in the X-ray spectrum. Therefore, when we look at the TGA thermogram, no mass loss is observed. Even with heating up to 800 °C, there is an absence of mass change. A similar interpretation is seen the DTA thermogram. All thermograms show a classic and routine Fe_3_O_4_ NPs slope decline [35]. The given DSC thermogram confirms the results of other thermal analyses. Moreover, it can be seen that the obtained nanoparticle structure has a high purity according to the DSC thermogram (Figure 2).

**Figure 3:**
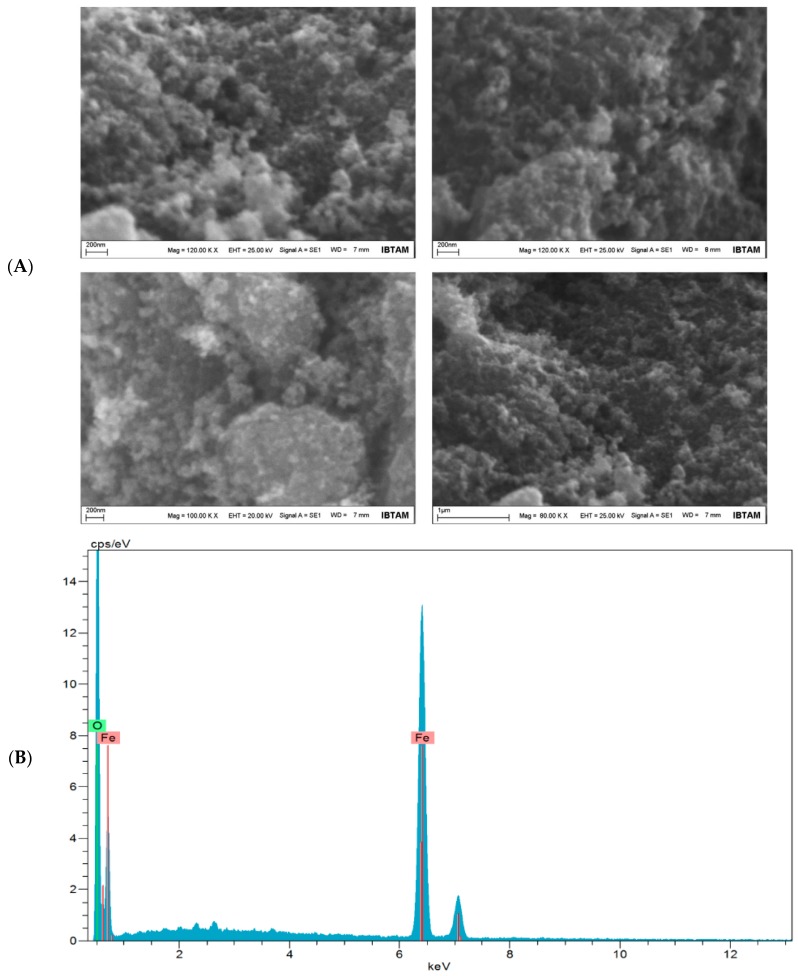
SEM images (**A**) and EDX spectrum (**B**) of the Fe_3_O_4_ NPs.

SEM images of Fe_3_O_4_ NPs are shown in Figure 3A. In these images, the NPs are very homogeneous and appear to be around 30 nm. Furthermore, electron diffraction spectroscopy revealed the Fe_3_O_4_ NPs structure. EDX images of Fe_3_O_4_ structures are given in Figure 3B. The basic values of O and Fe are clearly seen in this structure. For O and Fe, diffraction peaks are shown at 0.705 and 6.398 keV, respectively. This proves that the nanoparticles have a magnetic ferrite nanostructure.

### 3.2. Spermatozoon Kinematics

**Figure 4:**
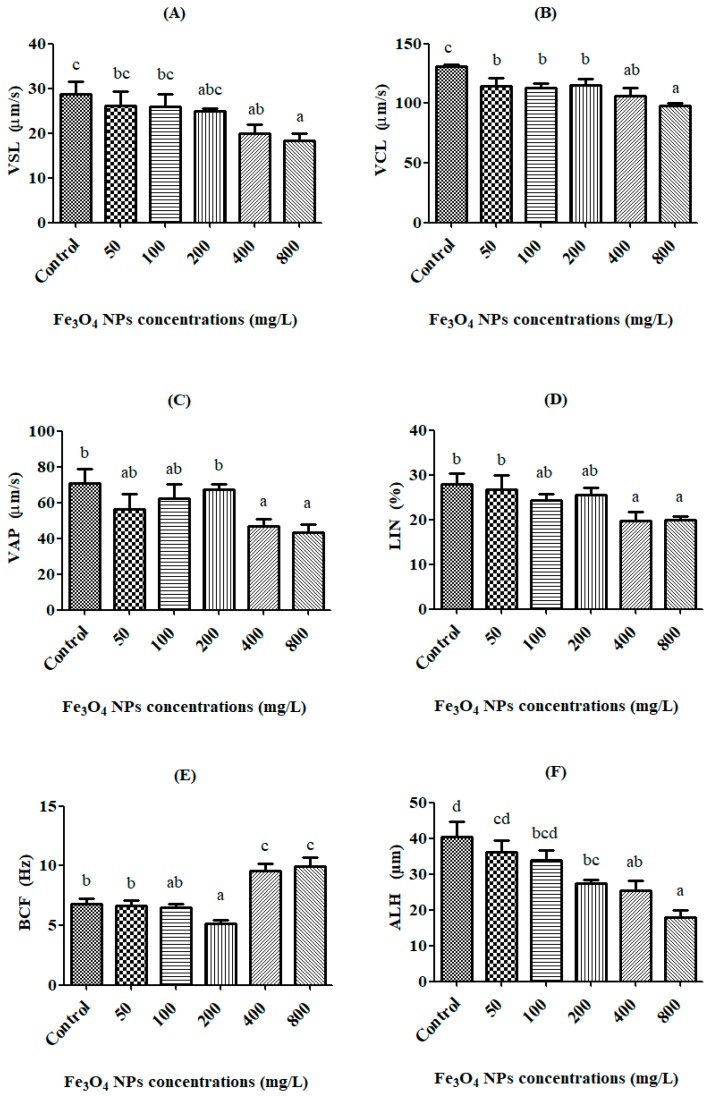
The exchanges of kinematics values on rainbow trout spermatozoon after exposure to Fe_3_O_4_ NPs. (**A**) VSL, straight line velocity (μm/s), (**B**) VCL, curvilinear velocity (μm/s), (**C**) VAP, angular path velocity (μm/s), (**D**) LIN, linearity (%), (**E**) BCF, beat cross frequency (cross/second), (**F**) ALH, amplitude of lateral displacement of the spermatozoa head (μm). Superscript alphabets ^(a,b,c,d)^ indicate significant differences among experimental groups (Means ± SE; *p* < 0.05).

The in vitro kinematics of rainbow trout spermatozoon exposed for 24 h to different concentrations (50, 100, 200, 400, and 800 mg/L) of Fe_3_O_4_ NPs are shown in Figure 4A–F. The VSL value decreased with increasing Fe_3_O_4_ NP doses. In particular, the decrease was found to be significant (*p* < 0.05) after exposure to 400 mg/L of Fe_3_O_4_ NPs (19.91 ± 1.93 μm/s) in Figure 4A. The VCL value of the control group was determined to be 130.84 ± 1.64 μm/s. The lowest VCL value was observed at the dose of 800 mg/L, being 97.88 ± 2.71 μm/s, and this decrease was also significant (*p* < 0.05) in Figure 4B. The control group had a VAP value of 70.76 ± 8.22 μm/s, which was the highest VAP value in all the groups. The changes in the VAP value were observed to be statistically significant (*p* < 0.05) at the doses of 400 mg/L and 800 mg/L of Fe_3_O_4_ NPs in Figure 4C.

The LIN value decreased with increasing Fe_3_O_4_ NPs doses, and this reduction was significant (*p* < 0.05) at both 400 mg/L and 800 mg/L doses in Figure 4D. The BCF value was observed to be significant (*p* < 0.05) at the dose of 400 mg/L of Fe_3_O_4_ NPs and up. The highest BCF value was 9.93 ± 0.76 Hz at the 800 mg/L dose in Figure 4E. The ALH value decreased with increasing Fe_3_O_4_ NPs doses, and this reduction was statistically significant (*p* < 0.05) after the 200 mg/L dose. The lowest ALH value was observed to be 17.82 ± 2.14 μm, at the 800 mg/L dose of Fe_3_O_4_ NPs in Figure 4F.

### 3.3. Oxidative Stress Biomarkers and the Effective Concentration (EC50)

**Figure 5:**
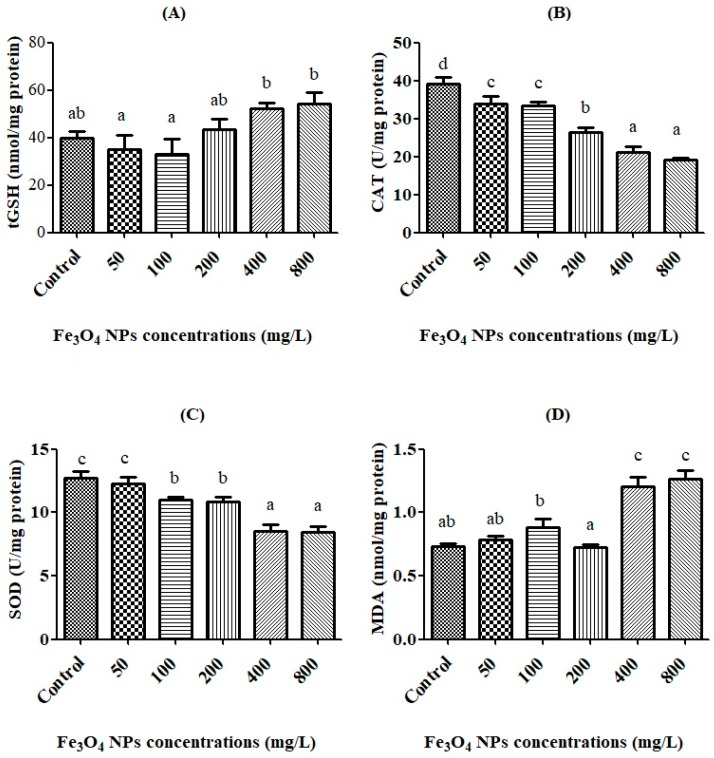
The exchanges of total glutathione (tGSH) (**A**), catalase (CAT) (**B**), superoxide dismutase (SOD) (**C**), and malondialdehyde (MDA) (**D**) values in rainbow trout spermatozoon after Fe_3_O_4_ NP exposure. Superscript alphabets ^(a,b,c,d)^ indicate significant differences among experimental groups (Means ± SE; *p* < 0.05).

The oxidative stress biomarkers (tGSH, CAT, SOD, and MDA) were determined after spermatozoon were exposed to Fe_3_O_4_ NPs (50, 100, 200, 400, and 800 mg/L) for 24 h, and the results were compared with the control group. The effect of Fe_3_O_4_ NPs on the biochemical parameters (tGSH, CAT, SOD, and MDA) are presented in Figure 5A–D. 

According to results, there was significant (*p* < 0.05) increase in tGSH levels after 400 mg/L dose (Figure 5A). On the other hand, as the dose of Fe_3_O_4_ NPs increased, the CAT activity decreased compared to the control value. This decrease was significant (*p* < 0.05) after the 50 mg/L dose of Fe_3_O_4_ NPs (Figure 5B). It was observed a significant (*p* < 0.05) decrease in SOD activity between the control and groups after a dose of 100 mg/L (Figure 5C). The level of MDA increased, but no significant (*p* ˃ 0.05) changes in the MDA levels were observed until the 200 mg/L dose. Only at doses of 400 and 800 mg/L significantly (*p* < 0.05) increased to the levels of MDA when compared to the control (Figure 5D).

**Figure 6:**
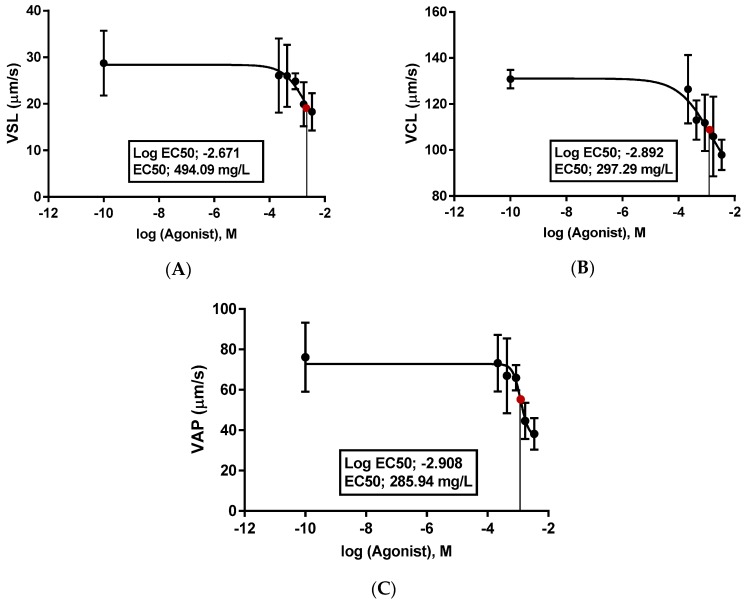
The exchanges of the EC50 values for VSL (straight line velocity) (**A**), VCL (curvilinear velocity) (**B**), and VAP (angular path velocity) (**C**) in rainbow trout spermatozoon after Fe_3_O_4_ NPs exposure.

The EC50 is the dose at which 50% of the maximum effect is produced, or the concentration of toxicant at which the toxicant is 50% effective. However, the effective concentration (EC50) against Fe_3_O_4_ NPs exposure was calculated in the values of VSL, VCL, and VAP of spermatozoon in rainbow trout (Figure 6A–C).

## 4. Discussion

The goal of the present study was to investigate the toxic impacts of different concentrations of Fe_3_O_4_ NPs on the spermatozoon of rainbow trout, *Oncorhynchus mykiss*. Although the in vitro spermatozoon toxicity has attracted the attention of many researchers, there are limited studies on the toxicity of nanoparticles for the risk assessment of nanomaterials, especially concerning fish reproductive systems.

The literature has shown that iron-based NPs have toxic effects on aquatic organisms. For example, Ates and his colleagues studied the chronic (60 days) effects of alpha and gamma Fe_2_O_3_ structures (0.1, 0.5, and 1.0 mg/L) on tilapia (*Oreochromis niloticus*). They focused on the effects of particle morphology on accumulation, elimination, hematology, and immune responses. According to their results, while the spleen had the largest accumulation, the intestine, kidney, liver, gills, brains and muscle tissues followed close behind. They determined no significant changes in hemoglobin, hematocrit, red blood cell, and white blood cell counts. While the level of serum glucose (GLU) decreased, the levels of glutamic oxaloacetic transaminase (GOT), glutamic pyruvic transaminase (GPT), and lactate dehydrogenase (LDH) increased. Finally, they determined that Fe_2_O_3_ NPs can induce differential uptake, assimilation, and immunotoxic effects on *O. Niloticus* under chronic exposure [12].

Another study focused on the effects of different doses (0.01, 0.1, and 1.0 mg/mL) of metal oxide NPs (SnO_2_, CeO_2_, and Fe_3_O_4_) on mortality and behavioral and biochemical responses of *Artemia salina* larvae. After 48 h of exposure, they determined that although these nanoparticles did not induce any mortality of the larvae, they caused changes in behavioral and biochemical responses. So, while the cholinesterase activities were found to be significantly decreased in the larva exposed to SnO_2_ NPs, they were significantly increased with CeO_2_ and Fe_3_O_4_ NPs. The glutathione-S-transferase (GST) activities decreased after exposure to all of the nanoparticles. The catalase activities gradually decreased in the SnO_2_ NPs, while there were no changes in CeO_2_ NPs. However, the catalase activities were significantly stimulated after exposure to all of the doses of Fe_3_O_4_ NPs [20].

Zhu et al. (2017) also studied the toxic effects of different doses (0, 25, 50, 100, 200, 400, and 600 mg/L) of Fe_3_O_4_ NPs on cysts and three larval stages of *Artemia salina*. They determined that Fe_3_O_4_ NPs caused a decrease in the body length of instar larvae. However, Fe_3_O_4_ NPs attached and caused irreversible damage to the gills and body surface of the larvae. Reactive oxygen species (ROS), malondialdehyde (MDA) content, total antioxidant capacity, and the activities of antioxidant enzymes (superoxide dismutase (SOD), catalase (CAT) and glutathione peroxidase (GSH)) were substantially increased following exposure to Fe_3_O_4_ NPs. They concluded with their study that Fe_3_O_4_ NPs have the potential to affect aquatic organisms when released into marine ecosystems [19].

Some researchers studied zebra fish sperm kinematics, fertilization success, and vitality by activated media with perfluorooctane sulfonate (PFOS) (0.09, 0.9, and 9 mg/L). They determined that PFOS decreased the percentage of motile sperm, the curvilinear velocity (VCL), and the mean angular displacement (MAD) of spermatozoa, but there was no effect on the straight line velocity (VSL) or the angular path velocity (VAP). Also, they observed a significant decrease in fertilization success of spermatozoon at doses of 0.9 mg/L PFOS or greater [36].

Linhartova et al. (2015) studied about the effects of different in vitro doses (0.5, 1.75, 2.5, 5, and 10 µg/L) of Tetrabrombisphenol A on DNA integrity and oxidative stress in Sterlet (*Acipenser ruthenus*) spermatozoon over 2 h. They found that the spermatozoon velocity and percent motile sperm were significantly decreased and the DNA were damaged after exposure to about a 2.5 µg/L dose. Also, while the thiobarbituric acid reactive substances (TBARS) level was significantly increased after 5 µg/L, CP and SOD activity were significantly increased after a 0.5 µg/L dose [37].

In our study, it was determined that the VSL and VCL values of spermatozoon were statistically significantly (*p* < 0.05) decreased after 24-h exposure to 400 mg/L of Fe_3_O_4_ NPs. Moreover, the VAP value was significantly decreased at the dose of 800 mg/L. So, Fe_3_O_4_ NPs decreased all velocities of rainbow trout spermatozoon. Our results are supported and paralleled by the findings of other researchers [36,37,38]. 

Gokduman et al. investigated the dose, treatment, and time-dependent toxicity of superparamagnetic Fe_3_O_4_ NPs (10 nm, 0–400 μg/mL) on primary rat hepatocytes. The obtained results suggested that the response of ROS increased with increasing concentrations of Fe_3_O_4_ NPs [39,40].

Biochemical parameters are important to determine of toxicities of Fe_3_O_4_ NPs in rainbow trout sperm spermatozoon. In our study, CAT and SOD activities decreased from 100 to 800 mg/L doses of Fe_3_O_4_ NPs. This inhibition in the enzyme activities was probably due to the overproduction of ROS, induced by Fe_3_O_4_ NPs. This is similar to the findings of Afifi et al. [41], in which the SOD and CAT activities were reduced in the brain of fish exposed to Ag NPs at a concentration of 4 mg/L. Similarly, CAT and SOD activities were reduced in the liver of ZnO NP-treated fish [42,43]. A significant increase in the tGSH level was observed after exposure to Fe_3_O_4_ NPs over 24 h. The level of MDA increased at doses of 400 and 800 mg/L of Fe_3_O_4_ NPs. The increase of MDA could be explained by the depletion of the antioxidant system. The same results have been reported previously in many studies. Adebayo et al. [44] and Afifi et al. [41] demonstrated the increasing effect of CeO_2_ and Ag NPs on the MDA level. Therefore, we decided that the balance between the oxidative stress and antioxidant system was broken and that oxidative damage in spermatozoon was caused by Fe_3_O_4_ NPs exposure, especially at doses of 400 and 800 mg/L. In this respect, the results are also similar to situations such as reproductive disorders and the deterioration of biological activities in rainbow trout spermatozoon. In addition, our results are supported by the data of other researchers [12,19,20,45,46].

## 5. Conclusions

According to our data, the kinematics (i.e., the velocities and movement styles) of rainbow trout spermatozoon were negatively affected by exposure to Fe_3_O_4_ NPs (50, 100, 200, 400 and 800 mg/L), for 24 h. The kinematics of spermatozoon exhibited significant (*p* < 0.05) decreases, which came into effect after 400 mg/L for VSL, VAP, and LIN, 50 mg/L for VCL, and 200 mg/L for BCF and ALH, as compared to the control group. However, Fe_3_O_4_ NPs have devastating effects on the antioxidant system after 50 mg/L for CAT and SOD, and 400 mg/L for MDA and tGSH, as compared to the control group. The effective concentration (EC50) against exposure to Fe_3_O_4_ NPs was calculated to be 494.09 mg/L for VSL, 297.29 mg/L for VCL, and 285.94 mg/L for VAP of spermatozoon in rainbow trout. Finally, we concluded that the balance between the oxidative stress and antioxidant system was broken and that oxidative damage was caused in spermatozoon fur to Fe_3_O_4_ NPs exposure, especially at concentrations of 200 mg/L and greater, in aquatic environments.

## References

[1] Naha P.C., Byrne H.J. (2013). Generation of intracellular reactive oxygen species and genotoxicity effect to exposure of nanosized polyamidoamine (PAMAM) dendrimers in PLHC-1 cells in vitro. Aquat. Toxicol..

[2] Naha P., Mukherjee S., Byrne H. (2018). Toxicology of Engineered Nanoparticles: Focus on Poly(amidoamine) Dendrimers. Int. J. Environ. Res. Public Health.

[3] Yang C.T., Li K.Y., Meng F.Q., Lin J.F., Young I.C., Ivkov R., Lin F.H. (2018). ROS-induced HepG2 cell death from hyperthermia using magnetic hydroxyapatite nanoparticles. Nanotechnology.

[4] Su C. (2017). Environmental implications and applications of engineered nanoscale magnetite and its hybrid nanocomposites: A review of recent literature. J. Hazard. Mater..

[5] Lei C., Sun Y., Tsang D.C.W., Lin D. (2018). Environmental transformations and ecological effects of iron-based nanoparticles. Environ. Pollut..

[6] Naha P.C., Al Zaki A., Hecht E., Chorny M., Chhour P., Blankemeyer E., Yates D.M., Witschey W.R.T., Litt H.I., Tsourkas A. (2014). Dextran coated bismuth-iron oxide nanohybrid contrast agents for computed tomography and magnetic resonance imaging. J. Mater. Chem. B.

[7] Teraphongphom N., Chhour P., Eisenbrey J.R., Naha P.C., Witschey W.R.T., Opasanont B., Jablonowski L., Cormode D.P., Wheatley M.A. (2015). Nanoparticle Loaded Polymeric Microbubbles as Contrast Agents for Multimodal Imaging. Langmuir.

[8] Chhour P., Gallo N., Cheheltani R., Williams D., Al-Zaki A., Paik T., Nichol J.L., Tian Z., Naha P.C., Witschey W.R. (2014). Nanodisco balls: Control over surface versus core loading of diagnostically active nanocrystals into polymer nanoparticles. ACS Nano.

[9] Gao L., Liu Y., Kim D., Li Y., Hwang G., Naha P.C., Cormode D.P., Koo H. (2016). Nanocatalysts promote Streptococcus mutans biofilm matrix degradation and enhance bacterial killing to suppress dental caries in vivo. Biomaterials.

[10] Liu Y., Naha P.C., Hwang G., Kim D., Huang Y., Simon-Soro A., Jung H.I., Ren Z., Li Y., Gubara S. (2018). Topical ferumoxytol nanoparticles disrupt biofilms and prevent tooth decay in vivo via intrinsic catalytic activity. Nat. Commun..

[11] Remya A.S., Ramesh M., Saravanan M., Poopal R.K., Bharathi S., Nataraj D. (2015). Iron oxide nanoparticles to an Indian major carp, *Labeo rohita*: Impacts on hematology, iono regulation and gill Na+/K+ATPase activity. J. King Saud Univ. Sci..

[12] Ates M., Demir V., Arslan Z., Kaya H., Yilmaz S., Camas M. (2016). Chronic exposure of tilapia (*Oreochromis niloticus*) to iron oxide nanoparticles: Effects of particle morphology on accumulation, elimination, hematology and immune responses. Aquat. Toxicol..

[13] Srikanth K., Trindade T., Duarte A.C., Pereira E. (2017). Cytotoxicity and oxidative stress responses of silica-coated iron oxide nanoparticles in CHSE-214 cells. Environ. Sci. Pollut. Res..

[14] Zhu X., Tian S., Cai Z. (2012). Toxicity Assessment of Iron Oxide Nanoparticles in Zebrafish (*Danio rerio*) Early Life Stages. PLoS ONE.

[15] Zhang Y., Zhu L., Zhou Y., Chen J. (2015). Accumulation and elimination of iron oxide nanomaterials in zebrafish (*Danio rerio*) upon chronic aqueous exposure. J. Environ. Sci..

[16] Zheng M., Lu J., Zhao D. (2018). Effects of starch-coating of magnetite nanoparticles on cellular uptake, toxicity and gene expression profiles in adult zebrafish. Sci. Total Environ..

[17] Kadar E., Tarran G.A., Jha A.N., Al-Subiai S.N. (2011). Stabilization of engineered zero-valent nanoiron with Na-acrylic copolymer enhances spermiotoxicity. Environ. Sci. Technol..

[18] Keller A.A., Garner K., Miller R.J., Lenihan H.S. (2012). Toxicity of Nano-Zero Valent Iron to Freshwater and Marine Organisms. PLoS ONE.

[19] Zhu S., Xue M.Y., Luo F., Chen W.C., Zhu B., Wang G.X. (2017). Developmental toxicity of Fe_3_O_4_ nanoparticles on cysts and three larval stages of *Artemia salina*. Environ. Pollut..

[20] Gambardella C., Mesarič T., Milivojević T., Sepčić K., Gallus L., Carbone S., Ferrando S., Faimali M. (2014). Effects of selected metal oxide nanoparticles on *Artemia salina* larvae: Evaluation of mortality and behavioural and biochemical responses. Environ. Monit. Assess..

[21] Mashjoor S., Yousefzadi M., Zolgharnain H., Kamrani E., Alishahi M. (2018). Organic and inorganic nano-Fe_3_O_4_: Alga *Ulva flexuosa*-based synthesis, antimicrobial effects and acute toxicity to briny water rotifer *Brachionus rotundiformis*. Environ. Pollut..

[22] Hatef A., Alavi S.M.H., Golshan M., Linhart O. (2013). Toxicity of environmental contaminants to fish spermatozoa function in vitro—A review. Aquat. Toxicol..

[23] Lahnsteiner F., Mansour N., Kunz F.A. (2011). The effect of antioxidants on the quality of cryopreserved semen in two salmonid fish, the brook trout (*Salvelinus fontinalis*) and the rainbow trout (*Oncorhynchus mykiss*). Theriogenology.

[24] Nynca J., Dietrich G.J., Dobosz S., Grudniewska J., Ciereszko A. (2014). Effect of cryopreservation on sperm motility parameters and fertilizing ability of brown trout semen. Aquaculture.

[25] Billard R. (1978). Some data on gametes preservation and artificial insemination in teleost fish. Actes Colloq..

[26] Özgür M.E., Balcıoğlu S., Ulu A., Özcan İ., Okumuş F., Köytepe S., Ateş B. (2018). The in vitro toxicity analysis of titanium dioxide (TiO_2_) nanoparticles on kinematics and biochemical quality of rainbow trout sperm cells. Environ. Toxicol. Pharmacol..

[27] Aebi H. (1984). Catalase in vitro. Methods Enzymol..

[28] Esrefoglu M., Akinci A., Taslidere E., Elbe H., Cetin A., Ates B. (2016). Ascorbic acid and beta-carotene reduce stress-induced oxidative organ damage in rats. Biotech. Histochem..

[29] Akerboom T.P.M., Sies H. (1981). Assay of Glutathione, Glutathione Disulfide, and Glutathione Mixed Disulfides in Biological Samples. Methods Enzymol..

[30] Bradford M.M. (1976). A rapid and sensitive method for the quantitation of microgram quantities of protein utilizing the principle of protein-dye binding. Anal. Biochem..

[31] Fauvel C., Suquet M., Cosson J. (2010). Evaluation of fish sperm quality. J. Appl. Ichthyol..

[32] Bordbar A.K., Rastegari A.A., Amiri R., Ranjbakhsh E., Abbasi M., Khosropour A.R. (2014). Characterization of Modified Magnetite Nanoparticles for Albumin Immobilization. Biotechnol. Res. Int..

[33] Loh K.S., Lee Y.H., Musa A., Salmah A.A., Zamri I. (2008). Use of Fe_3_O_4_ nanoparticles for enhancement of biosensor response to the herbicide 2,4-dichlorophenoxyacetic acid. Sensors.

[34] Catalano E., Di Benedetto A. (2017). Characterization of physicochemical and colloidal properties of hydrogel chitosan-coated iron-oxide nanoparticles for cancer therapy. J. Phys. Conf. Ser..

[35] Ulu A., Ozcan I., Koytepe S., Ates B. (2018). Design of epoxy-functionalized Fe_3_O_4_ @MCM-41 core–shell nanoparticles for enzyme immobilization. Int. J. Biol. Macromol..

[36] Xia J., Niu C. (2017). Acute toxicity effects of perfluorooctane sulfonate on sperm vitality, kinematics and fertilization success in zebrafish. Chin. J. Oceanol. Limnol..

[37] Linhartova P., Gazo I., Shaliutina-Kolesova A., Hulak M., Kaspar V. (2015). Effects of tetrabrombisphenol A on DNA integrity, oxidative stress, and sterlet (*Acipenser ruthenus*) spermatozoa quality variables. Environ. Toxicol..

[38] Gallo A., Manfra L., Boni R., Rotini A., Migliore L., Tosti E. (2018). Cytotoxicity and genotoxicity of CuO nanoparticles in sea urchin spermatozoa through oxidative stress. Environ. Int..

[39] Gokduman K., Bestepe F., Li L., Yarmush M.L., Usta O.B. (2018). Dose-, treatment- and time-dependent toxicity of superparamagnetic iron oxide nanoparticles on primary rat hepatocytes. Nanomedicine.

[40] Shaliutina O., Shaliutina-Kolešová A., Lebeda I., Rodina M., Gazo I. (2017). The in vitro effect of nonylphenol, propranolol, and diethylstilbestrol on quality parameters and oxidative stress in sterlet (*Acipenser ruthenus*) spermatozoa. Toxicol. In Vitro.

[41] Afifi M., Saddick S., Abu Zinada O.A. (2016). Toxicity of silver nanoparticles on the brain of *Oreochromis niloticus* and *Tilapia zillii*. Saudi J. Biol. Sci..

[42] Xiong D., Fang T., Yu L., Sima X., Zhu W. (2011). Science of the Total Environment Effects of nano-scale TiO_2_, ZnO and their bulk counterparts on zebra fish: Acute toxicity, oxidative stress and oxidative damage. Sci. Total Environ..

[43] Alkaladi A., El-Deen N.A.M.N., Afifi M., Zinadah O.A.A. (2015). Hematological and biochemical investigations on the effect of vitamin E and C on *Oreochromis niloticus* exposed to zinc oxide nanoparticles. Saudi J. Biol. Sci..

[44] Adebayo O.A., Akinloye O., Adaramoye O.A. (2018). Cerium oxide nanoparticle elicits oxidative stress, endocrine imbalance and lowers sperm characteristics in testes of balb/c mice. Andrologia.

[45] Song G., Gao Y., Wu H., Hou W., Zhang C., Ma H. (2012). Physiological effect of anatase TiO_2_ nanoparticles on Lemna minor. Environ. Toxicol. Chem..

[46] Meena R., Paulraj R. (2012). Oxidative stress mediated cytotoxicity of TiO_2_ nano anatase in liver and kidney of Wistar rat. Toxicol. Environ. Chem..

